# Prognostic Value of c-Myc Immunohistochemical Expression in Muscle Invasive Urothelial Carcinoma of the Urinary Bladder: A Retrospective Study

**DOI:** 10.31557/APJCP.2019.20.12.3735

**Published:** 2019

**Authors:** Amira Emad Elwy, Tarek Mohamed Elsaba, Ahmed Refaat Abd Elzaher, Mahmoud Ismail Nassar

**Affiliations:** 1 *Department of Pathology, *; 2 *Department of Medical Oncology, South Egypt Cancer Institute, *; 3 *Department of Pathology, Faculty of Medicine , Assiut University, Assiut, Egypt. *

**Keywords:** Urothelial carcinoma, muscle invasive, c-Myc, infiltrative pattern, prognosis

## Abstract

**Objective:**

This study aimed to investigate the immunohistochemical expression of c-Myc in muscle invasive urothelial carcinoma (MIUC) of the urinary bladder and to evaluate the correlation of c-Myc expression with different clinicopathological parameters and outcome, including a relatively new histopathological tumor characteristic that is the growth pattern of tumor invasion.

**Methods:**

A total of 66 formalin-fixed and paraffin-embedded sections of MIUC obtained from radical cystectomy specimens were enrolled. The sections were stained with c-Myc antibody using immunohistochemistry technique.

**Results:**

Tumor cells showed variability in nuclear c-Myc expression according to the growth pattern of invasion. The median H-score of nuclear expression of infiltrative pattern was significantly higher than that of non-infiltrative pattern (p<0.001). Nuclear expression of c-Myc in tumor tissue had a significant association with poor prognostic factors (sarcomatoid variant (p<0.001), perineural invasion (p=0.037), lymphovascular invasion (p<0.001), lymph node metastasis (p<0.001), distant metastasis (p=0.042) and advanced stage grouping (p=0.001). Kaplan Meier survival analysis demonstrated that c-Myc expression could not be significantly correlated with overall survival or disease free survival rates.

**Conclusion:**

Nuclear c-Myc seems to have a prominent role in epithelial to mesenchymal transition with consequential in tumor progression and metastasis, while it is not as much useful to predict the clinical behavior of patients with MIUC.

## Introduction

Bladder cancer (BC) is the 10^th^ most common cancer worldwide (Bray et al., 2018). It ranks higher among men, being the 6th most common cancer and the 9th cause of cancer related deaths (Bray et al., 2018). In Egypt, BC represents the 3rd most frequent cancer in both sexes (6.94%) and the 2nd in males (10.71%), based upon results of the National Population-Based Registry Program of Egypt 2008-2011 (Ibrahim et al., 2014). Egyptian males have a highest mortality rate (16.3 per 100,000) worldwide, which is twice to fourth as high as the rate in Europe and United States respectively (Jemal et al., 2011).

Urothelial carcinoma (UC) is the predominant histological type of BC, accounting approximately 90% (Miyazaki and Nishiyama, 2017). In Egypt, over the past few decades, there have been significant changes in the prevalence of the histological types of BC; classically, most of BC were squamous cell carcinoma (SCC) owing to schistosomiasis, whereas after 2000, UC is the most common (Salem and Mahfouz, 2012).

Muscle invasive urothelial carcinoma (MIUC) is a potentially lethal cancer with a 5-year relative survival rate ranges from less than 50% for stage II to less than 5% for stage IV (Flannery et al., 2018; Yousef and Gabril, 2018). Prognosis of MIUC is related to various pathological and clinical parameters and the most powerful prognostic factor is the pathological stage. Although these prognostic factors represent significant predictors, they do not provide accurate predictions for individual patients (Kucuk et al., 2015; Mitra, 2016).

Therefore, several studies in recent years are in attempts to determine new and reliable prognostic tools that can evaluate cancer aggressiveness, progression risk, probability of recurrence and overall prognosis, paving the way to a personalized cancer treatment to reduce morbidity and mortality (Cheng et al., 2014; Nagata et al., 2016).

There is no valid nuclear or cellular grade to stratify MIUC as opposed to SCC or adenocarcinoma. Based on that, Jimenez et al., (2000) displayed and recognized three main patterns of tumor invasion in MIUC of the urinary bladder into; nodular, trabecular and infiltrative in order to study whether architectural pattern could carry prognostic information equivalent to Gleason system for prostate carcinoma. They reported that an infiltrative growth pattern is associated with a dismal prognosis (Jimenez et al., 2000). Sequential studies also proved that infiltrative growth pattern has a large impact on pathological stage and may be a strong predictor of cancer specific survival (Kruger et al., 2004; Bircan et al., 2005; Langner et al., 2006; Denzinger et al., 2009; Otto et al., 2018).

c-Myc is a critical product of proto-oncogene located on the long arm of chromosome 8 (Kiemeney et al., 2008). It is a highly pleiotropic transcription factor protein exerting powerful regulation of vital cellular processes as cell growth, proliferation, differentiation and apoptosis (Sears, 2004). Deregulation of c-Myc plays a significant role in oncogenesis (Soucek and Evan, 2002). It is described by SoucekEvan (2002) as “the oncogene from hell”. c-Myc is documented to be deregulated in up to 70% of human cancers, leading to overexpression of the oncoprotein that can enforce most of the hallmarks features of cancer (Dang, 2012; Gabay et al., 2014).

The results of several studies reported that c-Myc is incriminated in pathogenesis of BC and suggested a significant association between overexpression of c-Myc protein and high tumor grade and advanced tumor stage (Mahdy et al., 2001; Watters et al., 2001; Watters et al., 2002; Zhao et al., 2015; Li et al., 2016). c-Myc is one of the molecular biomarkers that has been studied as a prognostic marker of BC since 90s until now, however controversies exist pertaining to its prognostic value in BC (Lipponen, 1995; Schmitz-Drager et al., 1997; Zaharieva et al., 2005; Massari et al., 2015; Li et al., 2016). 

In the present study, we aimed to evaluate c-Myc immunohistochemical expression in MIUC, correlate c-Myc expression with different clinicopathological parameters and assess its potential prognostic significance for these patients. Moreover, our study is the first to apply the association between c-Myc expression and a relatively new histopathological tumor characteristic that is the growth pattern of tumor invasion.

## Materials and Methods

*Patients and tissue specimens*

This retrospective study included 66 formalin-fixed and paraffin-embedded sections of MIUC of the bladder obtained from patients who had undergone radical cystectomy with pelvic lymphadenectomy at Surgical Oncology Department at South Egypt Cancer Institute during the period between January 2014 and December 2016. All specimens were retrieved from the archive of Pathology Department at South Egypt Cancer Institute. Clinical data were collected from the medical records and included: age, gender, distant metastasis, stage grouping, recurrence and 2-year survival data including overall survival (OS; calculated from the date of diagnosis to the date of death) and disease free survival (DFS; calculated from the date of operation to the date of the first observation of recurrence). All patients in this study did not receive neoadjuvant chemotherapy or radiotherapy.

The largest tumor diameter (cm) was obtained from the patients’ pathological reports. All hematoxylin and eosin (H and E) stained sections were re-evaluated independently by three pathologists to determine the variant and divergent differentiation of UC, presence of bilharziasis, perineural invasion (PNI), lymphovascular invasion (LVI), necrosis and pathological TNM stage. The tumors were re-staged according to American Joint Committee on Cancer (AJCC) staging system, 8^th^ edition (Magers et al., 2019) and histological variants were classified according to World Health Organization (WHO) classification of tumors of the urinary tract, 2016 (Humphrey et al., 2016). During the process of re-evaluation, three architectural patterns of tumor invasion were determined following the definition by (Jimenez et al., 2000). Morphologically the three patterns of tumor invasion defined as: (a) nodular pattern: arranged in variable sized, well-delineated, rounded nests of tumor cells with a tendency toward roundness maintained overall, (b) trabecular pattern: composed of broad trabeculae, anastomosing with each other and the trabeculae are at least three cells thick and (c) infiltrative pattern: consisted of narrow cords or single infiltrating cells and the cells are either small or highly pleomorphic. We considered the pattern of tumor invasion of sarcomatoid variant as infiltrative pattern. The percentage of each pattern was recorded for each tumor section. Images were captured with an Olympus Microscope BX43 using Toup-Cam Full HD Digital Camera (model number: XCAM1080PHB).

*c-Myc immunohistochemistry*

Formalin-fixed and paraffin-embedded tissue sections, each of 4-μm-thick, were cut from each block and stained immunohistochemically with c-Myc antibody ((A-14): sc-789, rabbit polyclonal affinity purified antibody raised against c-terminus of c-Myc protein of human origin, Santa Cruz Biotechnology, Santa Cruz, CA, USA) using the manufacturer’s protocol. Tissue sections were first incubated in the oven for 1 hour at 95^o^C, dewaxed with xylene and rehydrated with descending graded alcohol (100%-70%). For antigen retrieval, sections were immersed in an unsealed plastic container (Coplin jars) filled with diluted antigen retrieval solution using Dako EnVisionTM FLEX Target Retrieval Solution, Citrate buffer, Low PH 6.1 (50x) (Code DM829) and immersed in water bath at 90^o^C for 1 hour. After cooling at room temperature, sections were washed in distilled water for 2 minutes. The endogenous peroxidase was blocked using Dako EnVision^TM^ FLEX Peroxidase Blocking Reagent (Code SM801) for 5 minutes, then rinsed twice with diluted phosphate buffered saline (PBS) using Dako EnVisionTM FLEX Wash Buffer (20x) (Code DM831). The samples were incubated overnight (~20 hours) at 4°C with the primary antibody at dilution of 1:100 (optimum dilution). On the next day, the slides were washed with PBS twice. The slides were then treated with a secondary antibody applied for 20 min at room temperature using Dako EnVisionTM FLEX HRP (Horseradish peroxidase) (Code SM802) and again washed twice with PBS. Finally, the sections were visualized by diaminobenzidine staining using Dako EnVisionTM FLEX DAB and Chromogen (Code DM827) and Substrate Buffer (Code SM803), then the sections were counterstained with ready to use hematoxylin using Dako EnVisionTM FLEX Hematoxylin (Code SM806), dehydrated with gradient ethanol, cleared with xylene and sealed for microscopic examination. Burkitt lymphoma tissue sections served as positive and negative (by omitting the primary antibody) controls.

*Evaluation of c-Myc immunostaining*

The expression of c-Myc in each tumor section was assessed independently by 3 pathologists who had no previous knowledge of clinical data. Staining expression was assessed with regard to percentage of positive cells, cellular location and intensity. Histoscore (H-score) system was applied to quantify c-Myc expression with a range of 0-300. H-score was achieved by semi-quantitative assessment of both the intensity (classified as negative (0), weak (1+), moderate (2+) and strong (3+)) and the percentage of positive cells according to the following formula (Abdou et al., 2016):

H-score = 1 x (% cells 1+) + 2 x (% cells 2+) + 3 x (% cells 3+).

H-score was calculated for each pattern of tumor invasion (nodular, trabecular and infiltrative) and their summation gave H-score of the overall tumor.

*Statistical analysis*

Continuous variables were presented as mean ± standard deviation (SD) with median and range when appropriate, while categorical variables were presented as numbers and percentages. The Mann-Whitney U-test was used for comparing two independent groups and Kruskal Wallis test for more than two independent groups. Spearman’s rho correlation was used for measurement of strength and direction of association between two variables. Receiver Operating Characteristic (ROC) curve was used for selecting the best cut-off value for H-score of c-Myc nuclear expression of the overall tumor according to lymph node metastasis, to assess its potential significance as a prognostic biomarker in MIUC of the bladder. The patients’ survival distribution (OS and DFS) was calculated by Kaplan-Meier method and log rank test for comparing curves. A subsequent multivariate survival analysis was carried out according to the Cox proportional hazards regression model adjusting for potential confounding prognostic factors. All p values were two-tailed and considered statistically significant if ≤ 0.05. SPSS version 23.0 was used for data management and data analysis.

## Results

*Clinical and pathological characteristics*

**Figure 1:**
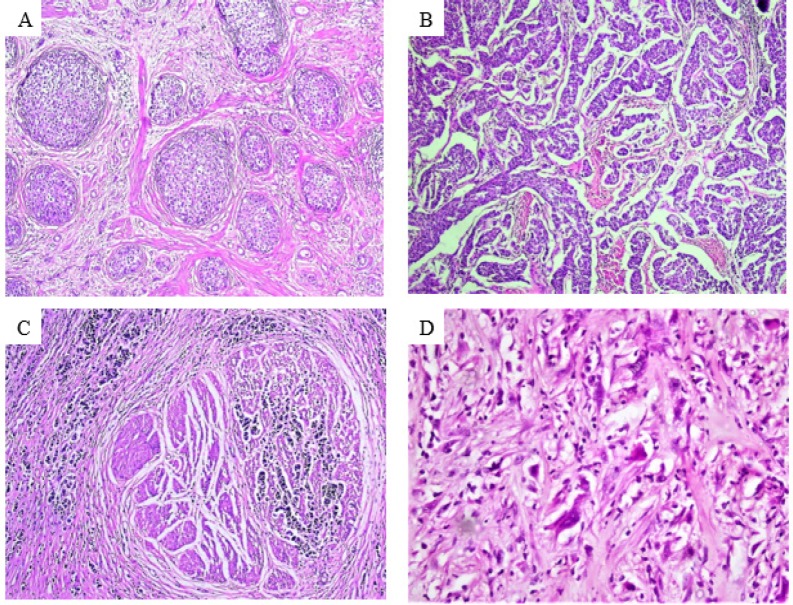
Growth Patterns of Tumor Invasion.(A) nodular pattern (x100), (B) trabecular pattern (x100), (C) infiltrative pattern of conventional urothelial carcinoma (x200) and (D) infiltrative pattern of sarcomatoid urothelial carcinoma (x 400).

The median age of patients was 60 year-old (range 38 to 89 year-old) for both gender. Sixty two cases (93.9%) were conventional UC and 4 (6.1%) sarcomatoid UC (3 out of 4 were pure sarcomatoid UC, while 1 was mixed with conventional UC). The tumor tissue in this study displayed one or more patterns of tumor invasion in the same tumor section. Nodular (Figure 1A), trabecular (Figure 1B) and infiltrative patterns (Figures 1C and 1D) were noted in 46, 60 and 43 tumor sections respectively. Two-year follow up data were available for 52 of 66 patients, while 14 cases were lost. Eight (12.1%) patients developed recurrence, 3 of them had local recurrence and 5 had distant metastasis to non-regional lymph nodes and/or distant organs. Total number of deaths at the end of the study was 17 (25.8%) out of 52 patients. Clinicopathological characteristics included in this study are summarized in (Table 1).

*Immunohistochemical profile of c-Myc*

**Figure 2:**
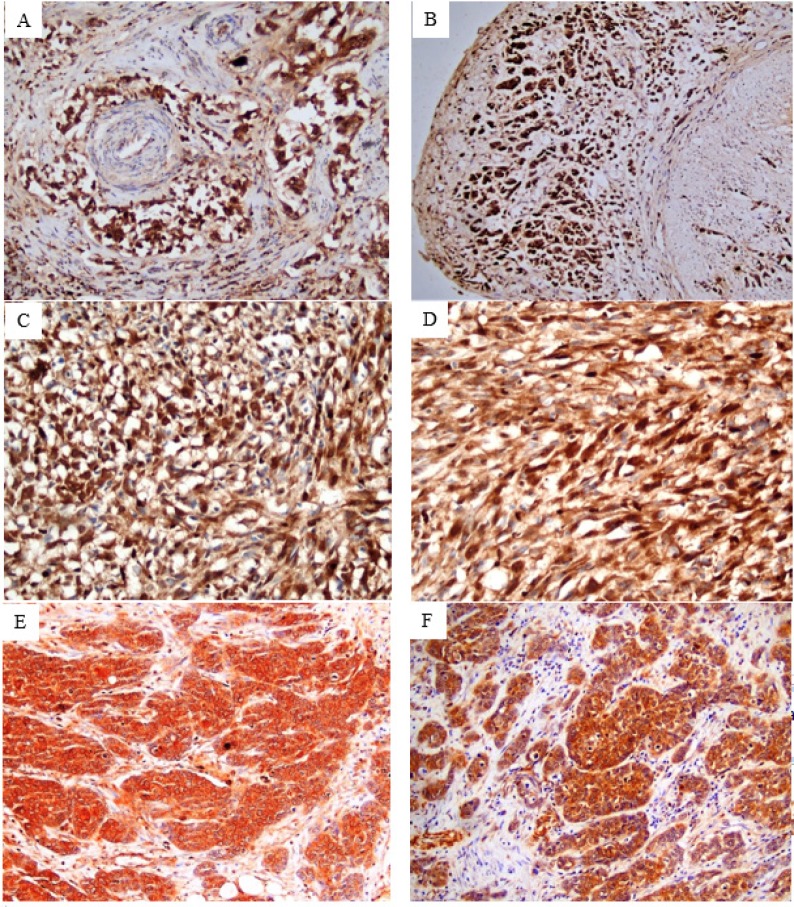
Tumor Cells Showed Variability in Nuclear Expression According to the Growth Pattern of Invasion. c-Myc immunostained sections revealed strong homogeneous nuclear expression in almost all tumor cells displayed infiltrative pattern of conventional urothelial carcinoma (x200) (A and B) and sarcomatoid urthelial carcinoma (x 400) (C and D), while sporadic tumor cells exhibited nodular and trabecular patterns showed heterogenous nuclear staining (x200) (E and F).

**Figure 3:**
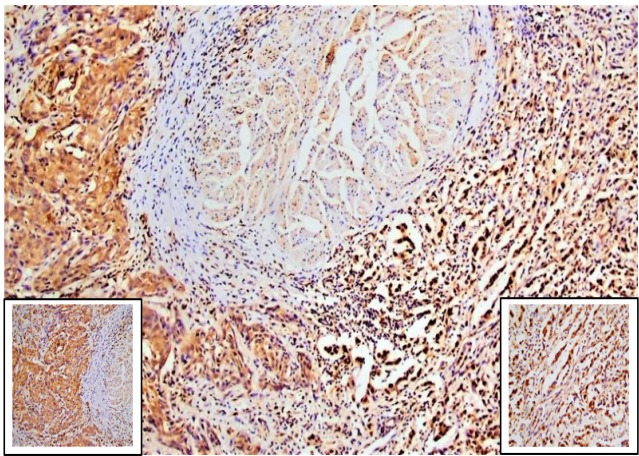
c-Myc Immunostained Section (x100) Revealed Strong Homogeneous Nuclear Staining in almost all Tumor Cells of Infiltrative Pattern (right side, x400), while sporadic tumor cells of trabecular pattern showed heterogeneous nuclear staining (left side, x400)

Normal urothelium adjacent to tumor showed weak to moderate cytoplasmic expression and negative nuclear staining. Stromal and endothelial cells showed focal cytoplasmic and nuclear expression. All 66 tumor sections showed positive immunoreactivity for c-Myc in the nucleus and the cytoplasm. Meanwhile, tumor cells showed variability in nuclear expression according to the growth pattern of invasion. Remarkably, almost all tumor cells of the 43 specimens displayed infiltrative pattern (including conventional and sarcomatoid UC) showed homogeneous strong nuclear staining (Figures 2A-2D and 3). However, sporadic tumor cells of 45 out of 60 specimens exhibited trabecular pattern and 15 out of 46 specimens of nodular pattern showed heterogeneous positive nuclear staining (Figures 2E, 2F and 3). H-scores of c-Myc expression in tumor tissue are summarized in (Table 2). Comparison between median H-scores of c-Myc nuclear expression of nodular, trabecular and infiltrative patterns was statistically significant (p<0.001). The highest median H-score was noted with the infiltrative pattern and the lowest one was noted with the nodular pattern. Comparison between median H-scores of cytoplasmic expression of nodular, trabecular and infiltrative patterns showed a borderline significance (p=0.070) (Table 3).

*Correlation between c-Myc immunohistochemical expression of the overall tumor and different clinicopathological characteristics*

The median nuclear expression was higher in sarcomatoid variant compared to conventional variant and the difference was statistically significant (p<0.001). High median nuclear expression was statistically significantly associated with PNI, LVI, lymph node metastases, distant metastasis and advanced stage grouping (p=0.037, p<0.001, p<0.001, p=0.042 and p=0.001) respectively. On the other hand, no statistical significance difference was noted in median cytoplasmic expression regarding different clinicopathological characteristics, except with variant of UC where the median cytoplasmic expression was statistically significance higher in sarcomatoid variant than in conventional variant (p=0.014) (Table 4).

*Survival analysis*

**Figure 4:**
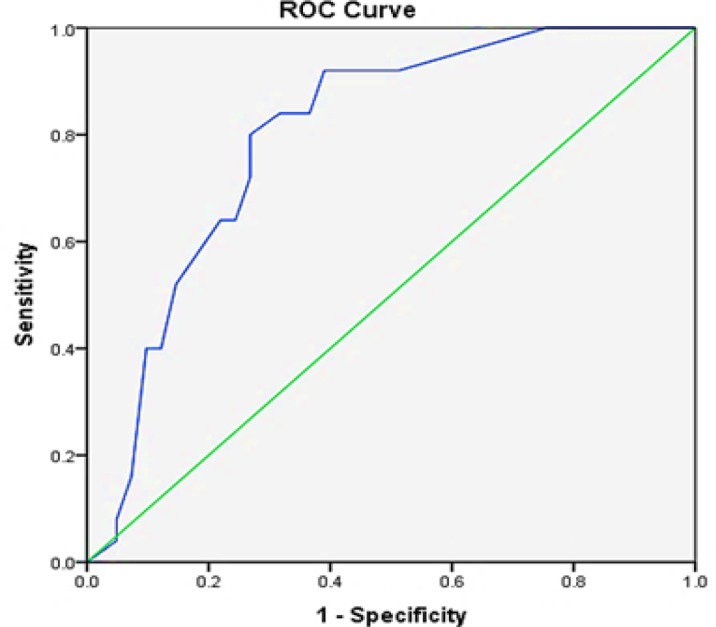
ROC Curve of H-score of Nuclear Expression of the Overall Tumor. The area under the curve (AUC) is 0.799.

ROC curve was used to determine a cut-off value in order to assess the potential significance of c-Myc IHC expression as a prognostic biomarker in MIUC of the bladder. ROC curve analysis revealed that H-score of nuclear expression of the overall tumor was robust in discriminating positive lymph nodes from negative ones with area under the curve (AUC) value 0.799 (95% CI 0.692 - 0.906) (asymptomatic significance < 0.001). ROC curve demonstrated that the best cut-off value of H-score of nuclear expression of the overall tumor was 120, by maximizing both sensitivity (true positive rate) and specificity (false positive rate) (Figure 4). However, H-score of cytoplasmic expression of the overall tumor was found to be non-discriminative on ROC curve, therefore its cut off point could not be defined.

**Figure 5:**
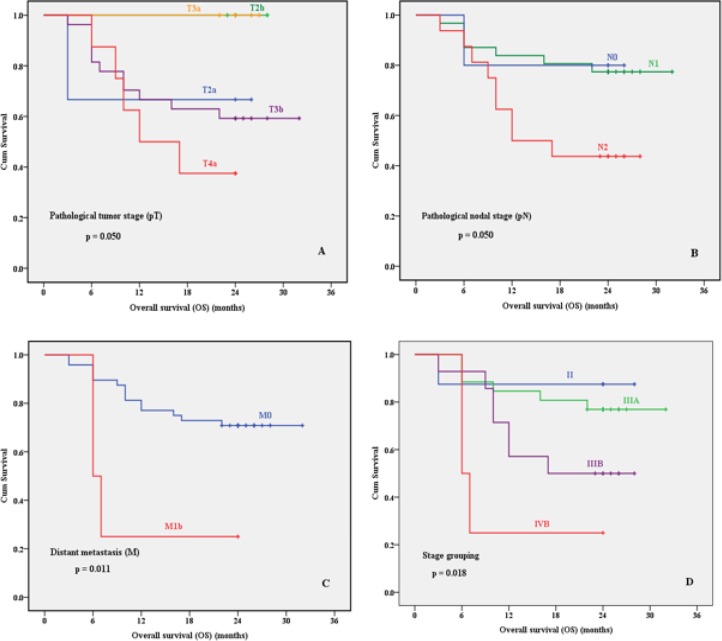
Kaplan Meier Survival Plots for Overall Survival of Patients Regarding Pathological Tumor Stage (pT) (A), Pathological Nodal Stage (pN) (B), Distant Metastasis Stage (M) (C) and Stage Grouping (D).

Kaplan Meier analysis revealed a significant association of advanced tumor stage (p=0.050), positive lymph nodes (p=0.050), distant metastasis (p=0.011) and advanced stage groups (p=0.018) with shorter 2-year OS (Figure 5). Other prognostic factors including c-Myc nuclear expression were not significantly correlated with 2-year OS or DFS rates (Table 5). Multivariate analysis for 2-year OS was applied for prognostic factors that were significant in Kaplan Meier analysis to adjust for confounder. Results revealed that no prognostic factor could be independent for 2-year OS (Table 6).

## Discussion

BC is a disease of remarkable heterogeneity in its clinical behavior. It is well known that the outcomes of patients with BC depend on different clinical and pathological factors, yet these factors do not always reflect the accurate prognosis. Therefore, studies are in a continuous search for accurate prognostic parameters (Knowles and Hurst, 2015; Kucuk et al., 2015). BC is one of the malignancies with a high molecular heterogeneity. Thus, a better understanding of the molecular features of BC will provide insightful information for individual tumor management and outcomes prediction (He et al., 2014; Knowles and Hurst, 2015). MIUC has a poor prognosis and is responsible for the majority of BC related deaths (Massari et al., 2015).

In cancer biology, c-Myc overexpression has been shown to be incriminated in the pathogenesis of various malignancies. However, its prognostic value in different cancers including BC remains a controversial issue. Several studies displayed significant association between the overexpression of c-Myc and poor prognosis in numerous tumors, while other studies revealed an opposite correlation. On the contrary, some reports did not support either of these conclusions (Zaharieva et al., 2005; Tsiatis et al., 2009; Massari et al., 2015; Zeng et al., 2015; Strindlund et al., 2017; He et al., 2018).

The present study was conducted to investigate whether c-Myc had a prognostic value for patients with MIUC of the bladder.

Based on knowledge that c-Myc is a transcription factor protein, so its nuclear localization in tumor cells was expected. The cytoplasmic localization for some unknown reason and of a controversial issue was also detected. Therefore, in this study we evaluated each expression separately and correlated each of them with different clinicopathological parameters to determine whether these patterns of localizations reflect a prognostic value on patients with MIUC.

Regarding the nuclear expression of c-Myc in tumor cells, interestingly we observed that almost all tumor cells displayed infiltrative pattern (including conventional and sarcomatoid UC) showed homogenous strong nuclear staining, whereas heterogeneous nuclear staining was noted in sporadic tumor cells exhibited trabecular and nodular patterns. Moreover, the median H-score of nuclear expression of the infiltrative pattern was statistically significant higher than that of trabecular and nodular patterns. 

To the best of our knowledge, there is no previous study described the relation between nuclear c-Myc expression and the histological growth pattern of tumor invasion in BC. High nuclear expression was noted in tumor cells of sarcomatoid UC and infiltrative pattern of conventional UC. This finding can be attributed to the role of c-Myc in epithelial to mesenchymal transition (EMT), based on the data reported by Cheng et al., (2011) that sarcomatoid variant represents the final pathway of BC dedifferentiation and EMT is responsible for this transformation. The infiltrative pattern of tumor invasion is also reported by Denzinger et al., (2009) to be due to loss of adhesion markers (e.g. E-cadherin), a phenomenon seen in EMT. It is stated that c-Myc promotes EMT by activation of the SNAIL transcription factor which is a strong repressor of E-cadherin protein (Wolfer and Ramaswamy, 2011). Supporting this explanation, CowlingCole (2007) who documented that c-Myc expression in human mammary epithelial cells induces a dramatic change in cell morphology with characteristic of EMT, and E-cadherin expression is repressed in cells expressing c-Myc. 

In our study, high c-Myc nuclear expression of the overall tumor was significantly associated with sarcomatoid variant, PNI, LVI, lymph node metastasis, distant metastasis and advanced stage grouping, while there was no significant association with gender, age, tumor diameter, divergent differentiation, bilharziasis, necrosis, pathological tumor stage (pT) and recurrence. 

The results of previous studies which investigated nuclear expression of c-Myc in MIUC of the bladder go in line with some results reported in our study. Lipponen (1995) assumed that positive c-Myc nuclear and/or cytoplasmic expression in UC did not significantly related to tumor stage (pT). Schmitz-Drager et al., (1997) also considered nuclear and/or cytoplasmic expression of c-Myc as positive in UC and proposed that c-Myc overexpression did not correlate with pT. Grapsa et al., (2014) as well considered c-Myc positive when >20% of tumor cells of UC showed nuclear and/or cytoplasmic staining and found that c-Myc did not correlate with pT-category. Contradicting our data, Zaharieva et al., (2005) documented that increased c-Myc copy number and *c-Myc *gene amplification were strongly associated with tumor stage (pT). In addition, Lipponen (1995) revealed that c-Myc nuclear expression did not be related to N and M pathological stage. Moreover, Fragkoulis et al., (2017) revealed that c-Myc negative expression in bladder cancer patients was associated with higher tumor stage, while the majority of c-Myc positive tumors were of low grade, non-muscle invasive and with negative lymph nodes.

Referring to other studies of c-Myc nuclear expression in malignancies of other organs, the results are highly variable. Green et al., (2016) revealed that positive c-Myc protein expression (nuclear and/or cytoplasmic) in breast cancer was significantly associated with tumor grade, lymph node stage and histological tumor type (medullary like tumors), while no significant association with tumor size or presence of lymphovascular emboli. Dueck et al., (2013) revealed that positive c-Myc nuclear staining in breast cancer was associated with nodal positivity and large tumor size. In prostatic cancer, Zeng et al., (2015) revealed that nuclear staining was associated with T-stage and distant metastasis. In esophageal SCC nuclear expression of c-Myc was significantly associated with poor differentiation, depth of invasion, lymph node metastasis and stage grouping (Lian et al., 2017). Sakr et al., Sakr et al., (2017) revealed a strong nuclear expression in undifferentiated carcinomas of thyroid than well-differentiated ones. All the findings of the previous studies go in line with our data regarding association of high c-Myc nuclear expression with PNI, LVI, lymph node metastases and advanced stage, hence, suggesting the role of c-Myc in tumor metastases and progression.

Regarding the prognostic role of c-Myc nuclear expression, the 2-year OS and DFS rates were not significantly different between low and high nuclear c-Myc groups. This result is in agreement with the results of previous studies done on UC of the bladder reported by Schmitz-Drager et al., (1997), Lipponen (1995) and Zaharieva et al., (2005), while discordant with Massari et al., (2015) who postulated that patients with MIUC displayed negative c-Myc nuclear expression had statistically significant better 2-year OS and DFS than those displaying positive expression. 

Concerning the cytoplasmic expression of c-Myc, careful review of the literature revealed that the mechanism of cytoplasmic expression of c-Myc remains an enigma.

Kotake et al., (1990) documented that positive cytoplasmic expression is due to the inappropriate conditions of fixation rather than biological cause. They studied the* IHC* expression of c-Myc in BC and reported that positive staining was observed in the cytoplasm not in the nucleus in formalin-fixed tissues. On the other hand, positive nuclear staining and negative cytoplasmic staining were observed in cryopreserved tissues. However, our results are mismatched with these data, as we applied c-Myc antibody on formalin-fixed and paraffin-embedded tumor tissue sections and both nuclear and cytoplasmic staining were noted. 

Royds et al., (1992) studied the staining pattern of c-Myc on colorectal cancer and adjacent colonic mucosa by both light and electron microscopes. Royds and colleagues clarified that cytoplasmic staining in tumor cells may be attributed to defects in c-Myc protein itself, its transportation from cytoplasm to the nucleus or both. The mechanisms of these defects may include: mutations in the *c-Myc *gene affecting the nuclear sequences, alterations in mRNA or protein turnover rates, defects in transportation of protein into the nucleus, defect interaction of c-Myc protein with nuclear binding factors or altered interactions of protein with cytoplasmic binding proteins. They reported that malignant tumor cells showed cytoplasmic staining and only nuclear blush. However, mucosa adjacent to the tumors showed cytoplasmic or pancellular expression. Ultrastructure patterns of staining showed that c-Myc associated with polyribosomes in the cytoplasm and dense chromatin in the nucleus. Moreover, c-Myc in tumor cells was very rarely noted in the nuclear pores in contrast to that of normal cells. 

Ciclitira et al., (1987) supposed that cytoplasmic localization of c-Myc could be due to cross-reaction of the antibody with an unrelated antigen which shared an epitope with the c-Myc protein.

Concerning the results of cytoplasmic expression in correlation with clinicopathological parameters, statistical data in our study revealed no significant results was noted between cytoplasmic expression of c-Myc and most of clinicopathological features (gender, age, largest tumor diameter, divergent differentiation, pattern of tumor invasion, bilharziasis, necrosis, PNI, LVI, TNM stage and stage grouping). However, there was significant high expression with sarcomatoid UC than conventional UC.

The results of other studies in literature which investigated the cytoplasmic expression of c-Myc in UC of the bladder go in line with our results regarding the tumor stage (T) (Lipponen, 1995; Schmitz-Drager et al., 1997; Grapsa et al., 2014).

Studies of cytoplasmic expression in malignancies other than UC exposed divergent results. Ruzinova et al., (2010) revealed that primarily cytoplasmic expression was associated with diffuse large B-cell lymphoma not harboring c-Myc translocation, while primarily nuclear or mixed nuclear and cytoplasmic expression was detected in aggressive B-cell lymphoma harboring c-Myc rearrangement. Geisler et al., (2004) reported that patients with endometrial carcinoma displaying cytoplasmic expression without nuclear staining had a better survival than those displaying combined nuclear and cytoplasmic expression. Cytoplasmic expression in breast cancer had been shown to be correlated with increased survival in a study done by Dueck et al., (2013). Gong et al., (2017) proposed that cytoplasmic expression was associated with high risk stratification of mantle cell lymphoma. He et al., (2014) demonstrated that high level of c-Myc cytoplasmic expression was significantly correlated with worse survival in patients with pancreatic cancer. Tselepis et al., (2003) observed discrete nuclear staining in Barret’s columnar metaplasia and as the epithelium became dysplastic, expression of c-Myc within the cytoplasm in addition to the nuclear staining was apparent to be accentuated in adenocarcinoma. Pennanen et al., (2018) concluded that strong cytoplasmic expression and weak nuclear expression in adrenocortical tumors associated with malignancy and short survival. 

According to the aforementioned data, we can establish that variability of the results between our study and different studies and among different studies each other does not seem to be related to the type of tumor, as both positive and negative correlations are detected in the same tumor. It looks like the difference in c-Myc expression and its correlation with various clinicopathological parameters may be related to diverse tumor tissue associated prognostic factors, different sample size, different approaches of detection of c-Myc and variable methods of interpretations of c-Myc expression. 

In conclusion, according to the results of the current study, nuclear c-Myc seems to have a prominent role in epithelial to mesenchymal transition with consequential in tumor progression and metastasis through its high expression in infiltrative pattern of invasion that is associated with EMT, while it is not as much useful to predict the clinical behavior of patients with MIUC of the bladder. Therefore, clinical trials with targeted anti-c-Myc therapies might provide additional ways for therapy of MIUC patients. However, large studies of patients with MIUC of the bladder with complete follow up are needed to validate this speculation. Study of c-Myc by methods other than immunohistochemistry is recommended to define its precise functional role in tumor cells and to solve the enigma of cytoplasmic localization.

**Table 1:** Clinicopathological Characteristics Included in this Study

Clinicopathological characteristics	Result *
Gender	
Males	50 (75.8%)
Females	16 (24.2%)
Age (y)	
Range	38 - 89
Median	60
Largest tumor diameter (cm)	
Range	1-10
Median	4
Variants of UC	
Conventional	62 (93.9%)
Sarcomatoid	4 (6.1%)
Divergent differentiation of UC	
Squamous differentiation	29 (43.94%)
Glandular differentiation	2 (3.03%)
Absent	35 (53.03%)
Pattern of tumor invasion	
Nodular pattern	46 (69.7%)
Trabecular pattern	60 (91%)
Infiltrative pattern	43 (65.2%)
Bilharziasis	
Present	39 (59.1%)
Absent	27 (40.9%)
Necrosis	
Present	33 (50%)
Absent	33 (50%)
PNI	
Present	61 (92.4%)
Absent	5 (7.6%)
LVI	
Present	45 (68.2%)
Absent	21 (31.8%)
Pathological tumor stage (pT)	
T2a	3 (4.5%)
T2b	11 (16.7%)
T3a	5 (7.6%)
T3b	36 (54.5%)
T4a	11 (16.7%)
N0	41 (62.1%)
N1	6 (9.1%)
N2	19 (28.8%)
Distant metastasis stage (M)	
M0	62 (93.9%)
M1b	4 (6.1%)
Stage grouping	
II	10 (15.2%)
IIIA	35 (53.0%)
IIIB	17 (25.8%)
Stage grouping	
IVB	4 (6.1%)
Patient follow up (2-year)	
Alive with disease	4 (6.1%)
Alive till recurrence of disease	8 (12.1%)
Alive without recurrence	40 (60.6%)
Alive at the end of the study	35 (53.0%)
Died at the end of the study	17 (25.8%)
Lost to follow up	14 (21.2%)
Overall survival (OS) (m)	
Range	3-32
Median	24
Disease free survival (DFS) (m)	
Range	1-30
Median	22

LVI, lymphovascular invasion; PNI, perineural invasion, UC, urothelial carcinoma; * Data are numbers (%) of cases unless otherwise specified.

**Table 2:** . H-scores of *c-Myc *Expression in Tumor Tissu

Parameter	Result
H-score of nuclear expression of the overall tumor	
Range	15 - 300
Median	110
Number of positive cases	66 of 66 (100%)
H-score of nuclear expression of nodular pattern	
Range	0 - 60
Median	0
Number of positive cases	15 of 46 (32.6%)
H-score of nuclear expression of trabecular pattern	
Range	0 - 90
Median	30
Number of positive cases	45 of 60 (75%)
H-score of nuclear expression of infiltrative pattern	
Range	30 - 300
Median	120
Number of positive cases	43 of 43 (100%)
H-score of cytoplasmic expression of the overall tumor	
Minimum	180 - 300
Median	270
Number of positive cases	66 of 66 (100%)
H-score of cytoplasmic expression of nodular pattern	
Range	20 - 220
Median	110
Number of positive cases	46 of 46 (100%)
H-score of cytoplasmic expression of trabecular pattern	
Range	20 - 230
Median	90
Number of positive cases	60 of 60 (100%)
H-score of cytoplasmic expression of infiltrative pattern	
Range	30 - 300
Median	120
Number of positive cases	43 of 43 (100%)

**Table 3:** Correlation between Patterns of Tuour Invasion and both c-Myc Nuclear and Cytoplasmic Expression

Pattern of tumor invasion	H-score of nuclear expression of each pattern of tumor invasion		H-score of cytoplasmic expression of each pattern of tumor invasion	
	Median	p value *	Median	p value *
Nodular pattern	0.00		110.00	
Trabecular pattern	30.00	< 0.001	90.00	0.070
Infiltrative pattern	120.00		120.00	

* p value by Kruskal Wallis test.

**Table 4:** Correlation between Different Clinicopathological Characteristics and both c-Myc Nuclear and Cytoplasmic Expression of the Overall Tumor

Clinicopathological characteristics	H-score of nuclear expression of the overall tumor		H-score of cytoplasmic expression of the overall tumor	
	Median/ r value	p value *	Median/ r value	p value *
Variant of UC		< 0.001		0.014
Conventional (N = 62)	100.00		270.00	
Sarcomatoid (N = 4)	300.00		300.00	
Differentiation of UC**		0.428		0.721
Squamous (N = 29)	110.00		280.00	
Absent (N = 35)	100.00		270.00	
Bilharziasis		0.733		0.524
Present (N = 39)	130.00		280.00	
Absent (N = 27)	110.00		260.00	
Necrosis		0.318		0.705
Present (N = 33)	90.00		270.00	
Absent (N = 33)	120.00		270.00	
PNI		0.037		0.990
Present (N = 61)	110.00		270.00	
Absent (N = 5)	60.00		280.00	
LVI		< 0.001		0.747
Present (N = 45)	130.00		270.00	
Absent (N = 21)	60.00		280.00	
pT stage		0.084		0.350
T2a (N = 3)	160.00		270.00	
T2b (N = 11)	60.00		280.00	
T3a (N = 5)	60.00		280.00	
T3b (N = 36)	105.00		260.00	
T4a (N = 11)	210.00		280.00	
pN stage		< 0.001		0.445
N0 (N = 41)	70.00		270.00	
N1 (N = 6)	185.00		275.00	
N2 (N = 19)	180.00		270.00	
M stage		0.042		0.229
M0 (N = 62)	100.00		270.00	
M1b (N = 4)	195.00		275.00	
Stage grouping		0.001		0.509
II (N = 10)	60.00		250.00	
IIIA (N = 35)	80.00		270.00	
IIIB (N = 17)	160.00		270.00	
IVB (N = 4)	195.00		275.00	
Recurrence		0.173		0.684
Present (N = 8)	95.00		260.00	
Absent (N = 40)	160.00		270.00	
Age (y)	-0.071	0.573	-0.059	0.638
Tumor diameter (cm)	0.142	0.257	0.084	0.500

LVI, lymphovascular invasion; N, total number of cases; PNI, perineural invasion; UC, urothelial carcinoma; * p value by Mann-Whitney U-test and Kruskal Wallis test for comparing two or more independent groups and by Spearman’s rho correlation for correlation between two ranked variables; ** Glandular group was excluded from analysis as they were only 2 cases.

**Table 5:** Kaplan Meier Analysis of Clinicopathological Characteristics and c-Myc Nuclear Expression for Overall Survival (OS) 52 Patients and Disease Free Survival (DFS) of 48 Patients with MIUC of the Bladder

Parameter	OS	DFS
	p value*	p value*
Gender	0.761	0.738
Males		
Females		
Variant of UC	0.409	0.449
Conventional		
Sarcomatoid		
Differentiation of UC**	0.453	0.367
Squamous		
Absent		
Pattern of tumor invasion		
Nodular pattern	0.463	0.644
Present		
Absent		
Trabecular pattern	0.695	0.837
Present		
Absent		
Infiltrative pattern	0.158	0.223
Present		
Absent		
Bilharziasis	0.285	0.270
Present		
Absent		
Necrosis	0.509	0.194
Present		
Absent		
PNI	0.624	0.766
Present		
Absent		
LVI	0.060	0.548
Present		
Absent		
pT stage	0.050	0.449
T2a		
T2b		
T3a		
T3b		
T4a		
pN stage	0.050	0.633
N0		
N1		
N2		
M stage	0.011	Not Done ***
M0		
M1b		
Stage grouping	0.018	0.800 ****
II		
Stage grouping	0.018	0.800 ****
IIIA		
IIIB		
IVB		
c-Myc nuclear expression	0.161	0.272
High (>120)		
Low (≤120)		

LVI, lymphovascular invasion; PNI, perineural invasion; UC, urothelial carcinoma; * p value by Log Rank test; ** Glandular group was excluded from analysis as they were only 2 cases; *** Not Done as the patients with M1b stage developed metastasis at the time of operation (i.e. had no disease free survival period), so no comparison analysis could be performed; **** Comparison was done between stages II, IIIA and IIIB. IVB did not included.

**Table 6:** Multivariate Overall Survival Analysis

Parameter	HR	95% CI	p value
Pathological tumor stage (pT)	1.873	0.823 - 3.090	0.106
Pathological nodal stage (pN)	2.087	0.570 – 7.632	0.266
Distant metastasis stage (M)	5.138	0.219 – 12.576	0.309
Stage grouping	0.570	0.048 - 5.314	0.570

CI, confidence interval; HR, Hazard ratio
